# Fine mapping of the *HLA* locus in Parkinson’s disease in Europeans

**DOI:** 10.1038/s41531-021-00231-5

**Published:** 2021-09-21

**Authors:** Eric Yu, Aditya Ambati, Maren Stolp Andersen, Lynne Krohn, Mehrdad A. Estiar, Prabhjyot Saini, Konstantin Senkevich, Yuri L. Sosero, Ashwin Ashok Kumar Sreelatha, Jennifer A. Ruskey, Farnaz Asayesh, Dan Spiegelman, Mathias Toft, Marte K. Viken, Manu Sharma, Cornelis Blauwendraat, Lasse Pihlstrøm, Emmanuel Mignot, Ziv Gan-Or

**Affiliations:** 1 Department of Human Genetics, McGill University, Montréal, QC Canada; 2 The Neuro (Montreal Neurological Institute-Hospital), McGill University, Montreal, QC Canada; 3 Stanford Center For Sleep Sciences and Medicine, Department of Psychiatry and Behavioral Sciences, Stanford University, Palo Alto, CA USA; 4 Department of Neurology, Oslo University Hospital, Oslo, Norway; 5 Institute of Clinical Medicine, University of Oslo, Oslo, Norway; 6 Department of Neurology and Neurosurgery, McGill University, Montréal, QC Canada; 7 Centre for Genetic Epidemiology, Institute for Clinical Epidemiology and Applied Biometry, University of Tübingen, Tübingen, Germany; 8 Department of Medical Genetics, University of Oslo and Oslo University Hospital, Oslo, Norway; 9 Department of Immunology, Oslo University Hospital, Oslo, Norway; 10 Laboratory of Neurogenetics, National Institute on Aging, National Institutes of Health, Bethesda, MD USA

**Keywords:** Parkinson's disease, Genomics

## Abstract

We fine mapped the leukocyte antigen (*HLA)* region in 13,770 Parkinson’s disease (PD) patients, 20,214 proxy-cases, and 490,861 controls of European origin. Four *HLA* types were associated with PD after correction for multiple comparisons, HLA-DQA1*03:01, HLA-DQB1*03:02, HLA-DRB1*04:01, and HLA-DRB1*04:04. Haplotype analyses followed by amino acid analysis and conditional analyses suggested that the association is protective and primarily driven by three specific amino acid polymorphisms present in most HLA-DRB1*04 subtypes—11V, 13H, and 33H (OR = 0.87, 95% CI: 0.83–0.90, *p* < 8.23 × 10^−9^ for all three variants). No other effects were present after adjustment for these amino acids. Our results suggest that specific *HLA-DRB1* variants are associated with reduced risk of PD, providing additional evidence for the role of the immune system in PD. Although effect size is small and has no diagnostic significance, understanding the mechanism underlying this association may lead to the identification of new targets for therapeutics development.

## Introduction

Although Parkinson’s disease (PD) is primarily a neurodegenerative disorder, the role of the immune system in the pathophysiology of PD is increasingly recognized based on animal and human studies^1–3^. The immune system can be involved in the initiation of PD, as well as in its progression, and that this involvement can be peripheral and central^3,4^.

Neuropathological studies have shown evidence for microglial activation in the brains of patients. However, it was initially unclear whether this activation was a part of the disease process, a consequence, or an epiphenomenon^5^. Genetic evidence also links the immune system with PD, since genes such as *LRRK2*, the human leukocyte antigen (*HLA*) locus, and possibly *BST1*, all associated with PD^6^ and have a role in the immune system^7–9^.

The *HLA* region on chromosome 6 includes genes that encode components of the major histocompatibility complex (MHC)^8^. Several genome-wide association studies (GWASs) have shown an association between the *HLA* locus and the risk of PD. In the latest GWAS, an association with *HLA-DRB5* has been reported, with a potential effect of the rs112485576 single nucleotide polymorphism (SNP) on the expression of *HLA-DRB5*^6^. Previous studies have suggested different associations with *HLA-DQA2*, *HLA-DQB1*, *HLA-DRA*, *HLA-DRB1*, *HLA-DRB5*, and with haplotypes within the *HLA* region in Europeans^10–15^.

In this study, we performed HLA alleles, haplotypes, and amino acid analyses in PD on 12,137 patients, 14,422 proxy patients, and 351,953 controls. We further performed conditional analyses to fine map and identify specific drivers of the association with PD in the *HLA* region.

## Results

### Meta-analysis of HLA types in PD suggests a single association

**Fig. 1:**
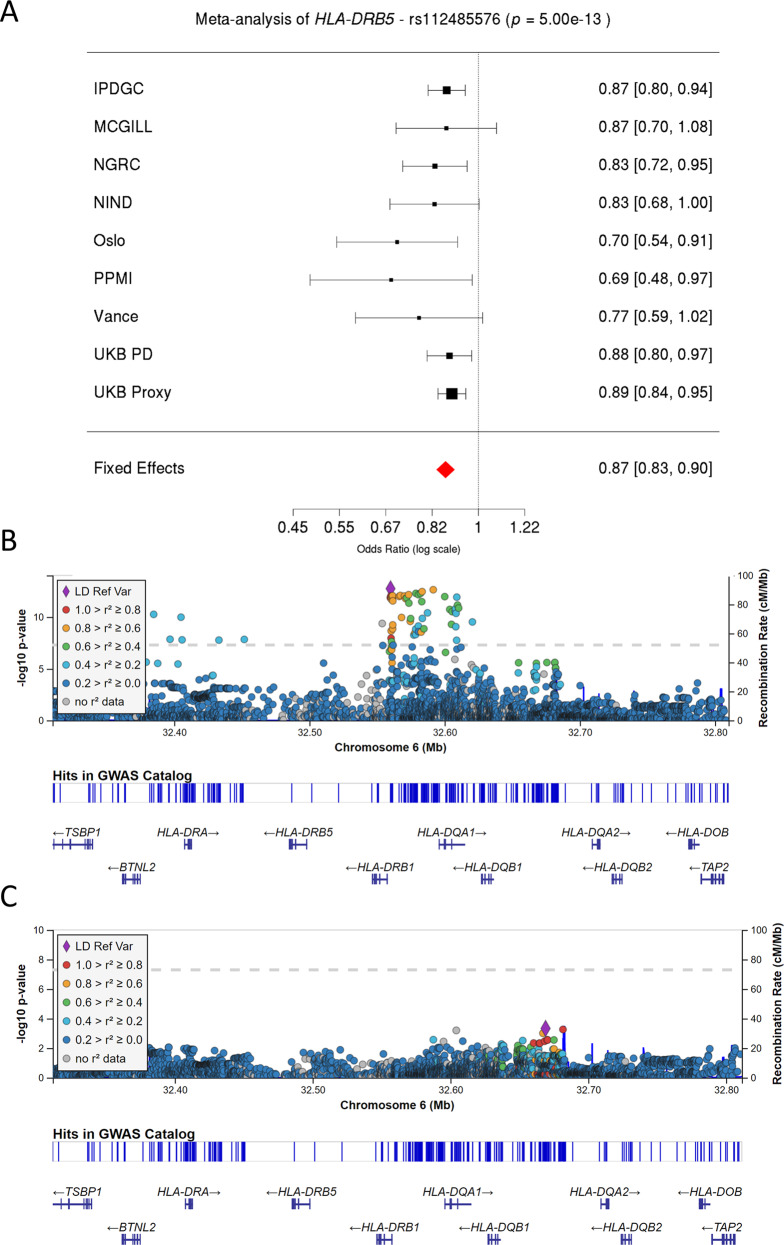
Validation of previously associated top *HLA* locus SNP (rs112485576) in our cohort. **A** Forest plot describing the effect size and 95% confidence interval of rs112485576 for each cohort and fixed-effect meta-analysis. **B**, **C** Two LocusZoom plots highlighting the significant variants before (**B**) and after (**C**) the conditional analysis on rs112485576. Dashed lines correspond to the significance threshold. Linkage disequilibrium values are shown with respect to the most significant SNP in the locus.

After standard QC, a total of 12,137 patients, 14,422 proxy patients, and 351,953 controls were included in the analysis (Supplementary Data 1 details the number of individuals analyzed from each cohort for each allele, and Supplementary Data 2 provides basic demographic information for each cohort). As shown in Fig. 1A, our SNP-level meta-analysis identified the previous association for SNP, rs112485576, in the *HLA* locus (in the current analysis: OR = 0.87, 95% CI: 0.83–0.90, *p* = 5.00 × 10^−13^, in the previous meta-analysis: OR = 0.85, 95% CI: 0.82–0.87, *p* = 6.96 × 10^−28^)^6^. No other HLA SNPs were significantly associated with PD after adjusting for rs112485576 (Fig. 1B, C), indicating that the association of this locus was primarily driven by a single genetic risk factor. We also identified the previous associations for the SNPs rs17425622, rs2395163, rs3129882, and rs9275326 in the *HLA* locus (Supplementary Figs. 1–4). All these SNPs were reported in different previous studies, are in LD, and all represent the same main haplotype that we report in the current analysis.

We next performed a meta-analysis association study of all *HLA* types with carrier frequency above 1%. After *HLA* imputation, a total of 141 different *HLA* types across 10 *HLA* loci were included (setting the Bonferroni corrected threshold for statistical significance on *α* = 3.55 × 10^−4^; 0.05/141). Following these analyses, we found four *HLA* alleles that were associated with PD (Table 1, results for other *HLA* alleles are detailed in Supplementary Data 3): HLA-DQA1*03:01, HLA-DQB1*03:02, HLA-DRB1*04:01, and HLA-DRB1*04:04. These four alleles are all located within a small genomic segment and have similar odds ratios ranging between 0.84 and 0.89. Three of the four alleles have similar carrier frequencies, indicating that they could be part of the same haplotypes, with the fourth potentially representing a sub-haplotype (Table 1).

**Table 1:** Meta-analyses of HLA alleles association.

Allele	Freq cases	Freq controls	OR (95% CI)	*P* value^1^	Direction	HetPVal
DQA1*03:01	0.168	0.185	0.86 (0.81-0.90)	3.54e−08	–	0.85
DQB1*03:02	0.182	0.199	0.87 (0.83-0.92)	7.62e−07	–	0.58
DRB1*04:01	0.187	0.221	0.89 (0.84-0.94)	2.82e−05	–	0.87
DRB1*04:04	0.068	0.082	0.84 (0.77-0.91)	8.21e−05	–	0.85

*Freq cases* carrier frequency of allele in patients, *Freq controls* carrier frequency of an allele in controls, *OR (95% CI)* odds ratio and 95% confidence interval, *Direction* direction of beta for each cohort, *HetPVal* *P* value of heterogeneity.

^1^Bonferroni correction for multiple comparisons sets the threshold for statistical significance to *α* = 3.55 × 10^−4^.

### HLA haplotype analysis

For haplotype analysis, we allowed for up to three genes to be included (Table 2) in each haplotype, since including more than three genes generated multiple haplotypes with low allele frequency that could not be analyzed at the current sample size. A total of 84 different HLA haplotypes (Supplementary Data 4) with allele frequency >1% were identified, setting the cut-off Bonferroni corrected *p* value for statistical significance at *α* = 5.95 × 10^−4^. Three different HLA haplotypes were associated with PD after correction for multiple comparisons: DQA1*03:01–DQB1*03:02, DRB1*04:01–DQA1*03:03, and DRB1*04:04–DQA1*03:01. Upon further examination, this association was found to be driven by several well-known sub-haplotypes, DRB1*04:04–DQA1*03:01–DQB1*03:02, and DRB1*04:01–DQA1*03:01/3–DQB1*03:01/2 (Supplementary Data 4). Because both DQA1*03:01 and DQA1*03:03 as well as DQB1*03:01 and DQB1*03:03 are present within the extended DRB1*04:01 haplotype, it is likely that these associations are driven by *DRB1*.

**Table 2:** Meta-analyses of HLA haplotype association.

Haplotype	Freq cases	Freq controls	OR (95% CI)	*P* value^1^	Direction	HetPVal
DQA1*03:01–DQB1*03:02	0.157	0.173	0.87 (0.82–0.93)	7.21e−05	++	0.62
DRB1*04:04–DQA1*03:01	0.067	0.081	0.83 (0.76–0.91)	9.82e−05	+	0.34
DRB1*04:01–DQA1*03:03	0.115	0.143	0.87 (0.81–0.94)	4.96e−04	+	0.71

*Freq cases* carrier frequency of a haplotype in patients, *Freq controls* carrier frequency of a haplotype in controls, *OR (95% CI)* odds ratio and 95% confidence interval, *Direction* direction of beta for each cohort, *HetPVal* *P* value of heterogeneity.

^1^Bonferroni correction for multiple comparisons sets the threshold for statistical significance to *α* = 5.95 × 10^−4^.

### Meta-analysis of the association of HLA amino acid changes with PD

To further identify the specific source of the association in the *HLA* locus, we performed an analysis of 636 amino acid changes in the HLA genes, setting the cut-off Bonferroni corrected *p* value for statistical significance at *α* = 7.86 × 10^−5^. Ten amino acid changes were significantly associated with a reduced risk of PD (Supplementary Data 5). The top three associated variants are linked amino acids 11V, 13H, and 33H (Table 3) present in all DRB1*04 subtypes, complementing the HLA haplotype analysis. Four other variants, 26S, 47Q, 56R, and 76V, in the *DQA1* gene, are in perfect LD with each other (*r*^2^ = 1, *D*′ =1, Supplementary Data 5) and in partial LD (*r*^2^ = 0.38, *D*′ = 0.85) with the *DRB1* variants. The association of these *DQA1* variants is weaker than the *DRB1* variants in terms of both effect size and statistical association (Supplementary Data 5). Three other variants, 71T, 74E, and 75L, are in the *DQB1* gene, are also in perfect LD with each other (*r*^2^ = 1, *D*′ = 1, Supplementary Data 5) and in partial LD (*r*^2^ = 0.16, *D*′ = 0.99) with *DRB1* 13H and 33H. As an additional quality control step, we repeated all the analyses reported above without the proxy cases. In these analyses, the results did not change, with all associations having the same direction of effect and with the same magnitude. No additional associations were found.

**Table 3:** Meta-analyses of HLA amino acid changes association.

Amino acid	Freq cases	Freq controls	OR (95% CI)	*P* value^1^	Direction	HetPVal
*DRB1* 13H	0.289	0.331	0.87 (0.83–0.91)	4.32e−09	–	0.60
*DRB1* 33H	0.289	0.331	0.87 (0.83–0.91)	4.32e−09	–	0.60
*DRB1* 11V	0.302	0.342	0.87 (0.83–0.91)	8.22e−09	–	0.45

*Freq cases* carrier frequency of an amino acid in patients, *Freq controls* carrier frequency of an amino acid in controls; *OR (95% CI)* odds ratio and 95% confidence interval, *Direction* direction of beta for each cohort, *HetPVal* *P* value of heterogeneity. *P* value of heterogeneity.

^1^Bonferroni correction for multiple comparisons sets the threshold for statistical significance to *α* = 7.86 × 10^−5^.

### Conditional analyses confirm that DRB1*04 amino acid variants likely drive the association of the *HLA* locus with PD

To further determine the specific genes or variants that drive these associations, we performed a set of conditional analyses and re-analyzed the allele types, haplotypes, and amino acid associations with PD. We conditioned the HLA type regression model on the following: First, we performed the conditional analysis using the top hit from the most recent PD GWAS, rs112485576, to examine the LD effect between this SNP and the different allele and amino acid associations reported in the current study. We further conditioned the HLA type regression models on DQA1*03:01, DQA1*03:03, and the *DRB1* variant 13H. We have also adjusted for the PD PRS, to examine a potential polygenic effect (Supplementary Data 3 and 5). While the adjustment for the *DRB1* variant 13H completely eliminated the associations in the *DQA1* gene, adjustment for DQA1*03:01 and DQA1*03:03 did not completely eliminate the association of the *DRB1* gene (Supplementary Data 3), again supporting this gene and these specific amino acids (11V, 13H, and 33H, Table 3) as the drivers of the association in the *HLA* locus. Adjustment for PRS did not change the results. It is also worth noting that the *DRB1* variants 11V (*r*^2^ = 0.96, *D*′ = 0.99), 13H (*r*^2^ = 0.99, *D*′ = 0.99), and 33H (*r*^2^ = 0.99, *D*′ = 0.99) are in LD with rs112485576, the top GWAS hit in this locus.

## Discussion

**Fig. 2:**
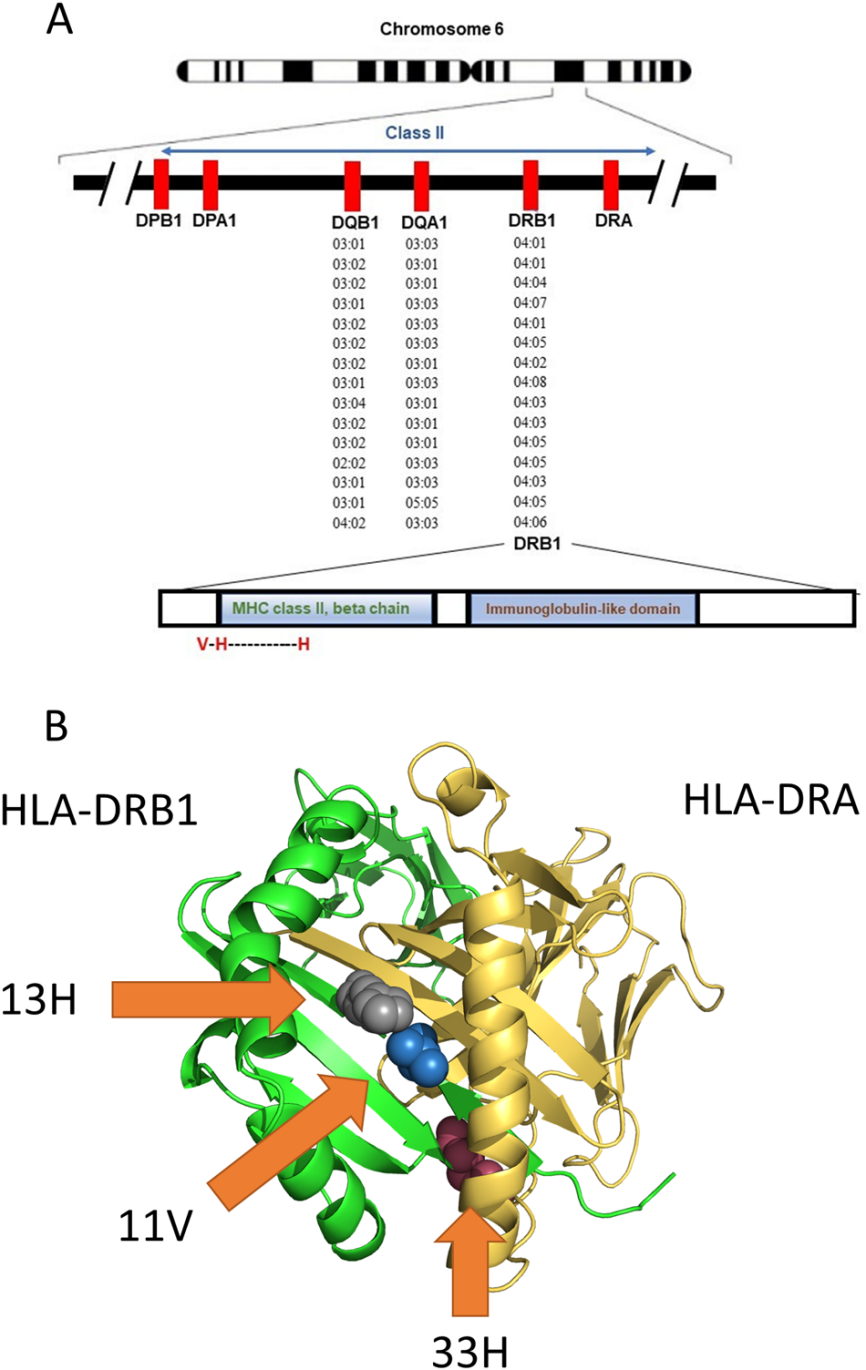
Association of the *HLA-DRB1* alleles and location of associated amino acids. **A** The location of the HLA locus, alleles, and amino acids associated with Parkinson’s disease in the current study. **B** 3D model of HLA-DRB1–HLA-DRA and the location of the 11V, 13H, and 33H amino acids associated with PD (highlighted by arrows). The model was generated with PyMol v. 2.4.1 (pdb 4is6).

In the current study, we performed a thorough analysis of the *HLA* region and examined its association with PD in the European population using a total of 12,137 patients, 14,422 proxy patients, and 351,953 controls. The importance of the current study lies in its size and comprehensive approach (including validation of imputation in over 3500 individuals), which allowed us to confirm previously reported associations but also refute with a high degree of confidence other reported associations. Following a series of regression models and conditional analyses, our results indicate that the drivers of the association in the *HLA* region are three amino acid changes specific of HLA-DRB1*04 subtypes, 11V, 13H, and 33H (Fig. 2). Two of these amino acid changes, 13H and 33H are in perfect LD, and 11V is in very strong LD with the other two variants. This study agrees with a smaller HLA sequencing study^12^ in 1597 PD cases and 1606 controls which also observed a protective effect of DRB1*04 and the same amino acids, although it also reported additional associations with DRB1*01:01 and DRB1*10:01 which were not confirmed in the current study. Interestingly, the V–H–H motif at positions 11V, 13H, and 33H are central to the DRB1*04:01 heterodimer and contribute to peptide binding, notably through pocket P6^16^ (Fig. 2).

Previous studies on the *HLA* genomic region in PD have reported associations of different genes and HLA types with PD, including *HLA-DQA2*, *HLA-DQB1*, *HLA-DRA*, *HLA-DRB1*, and *HLA-DRB5*^10–15^. The largest of these studies which performed proper HLA type analysis, included 7,996 cases and 36,455 controls, compared to the current study including more than 24,000 cases and 350,000 controls, with only partial overlap of samples (more details on these and other studies are in Supplementary Data 6). Therefore, the current study is more powered to detect true positive associations and true negative results, although we cannot rule out small effects that could not be detected even in our larger sample size. Most of the previously reported associations were not replicated in the current, larger study, which allows us to focus more on the associations that were confirmed. The suggested association with *HLA-DRB5* was reported in the most recent PD GWAS, in which no proper HLA imputation and analysis were performed. This reported association is based on an expression quantitative trait locus analysis, as the top associated SNP in this region, rs112485576 was also associated with differential expression of *HLA-DRB*^6^. A previous study of 2000 PD patients and 1986 controls has implicated a non-coding variant (rs3129882) within *HLA-DRA* as driving the association with PD and suggested that this variant affects the expression of *HLA-DR* and *HLA-DQ* genes^11^. Similarly, another study suggested that the same variant in *HLA-DRA* (rs3129882) is associated with differential expression of MHC-II on immune cells^17^. While our study does not rule out this possibility, since the main variants driving the association are amino acid changes in DRB1*04 that will affect epitope binding ability, it is likely that the effect on PD risk is through these variants and not due to modified expression. Additional functional studies will be required to study this hypothesis.

The current study adds further support to the hypothesis suggesting an involvement of the peripheral and central immune systems in PD. On top of the *HLA* locus, several other genes with potential roles in the immune system, including *LRRK2* and potentially *BST1*^7,9^, have been implicated in PD^6^. In the periphery, there are notable changes in the immune system of PD patients compared to controls, as peripheral monocytes have differential expression of immune-related proteins and markers^3^. Whether these changes are drivers of the disease or a result of the disease is still undetermined, but accumulating evidence suggests that they can be part of the pathogenic process of PD. In the central nervous system, pathological studies suggest that microglial cells may have a central role in PD^18^. Microgliosis is a prominent pathological finding in the post-mortem brains of PD patients, and evidence suggests that microglial activation occurs early in the disease process and may be involved in the pathogenesis of PD^3^. The specific contribution of *HLA* to these processes is still unclear and needs to be further studied.

One intriguing possibility that may directly involve *HLA* with PD is the potential interaction of HLA-DRB1*04 with α-synuclein, notably an epitope surrounding p.S129. Recent data has shown that α-synuclein fragments can bind MHC and increase T cell reactivity^19^. This activity is proinflammatory, involves both CD4 and CD8 cells, and may occur before the onset of motor symptoms^19,20^, suggesting the involvement of inflammation in early PD pathogenesis. Studying specific α-synuclein fragments has suggested that two major regions of α-synuclein may be associated with increased T cell reactivity in PD, with preferential CD4 activity: an N-terminal region involving amino acid p.Y39 and a C-terminal region surrounding amino acid p.S129, two important residues undergoing phosphorylation^19^. The phosphorylation of p.S129 is particularly interesting as it is well known for promoting aggregation^21,22^. Further analysis focused on the p.Y39 region suggested an association with α-synuclein-specific p.Y39 T cell responses and HLA DRB1*15:01 and DRB5*01:01 presentation. This association was abolished by phosphorylation, which reduced the binding of p.Y39-phosphorylated α-synuclein^19,20^. Other experiments by these authors have also suggested CD8+ T cells responses mediated by HLA-A11*01 presentation of epitopes in the same N-terminal region of α-synuclein. However, in the current study, we could not confirm an association of these HLA types with PD.

More interestingly in the context of our work, increased CD4+ T cell response to both p.S129 phosphorylated and unphosphorylated α-synuclein was also demonstrated, suggesting the involvement of DQB1*05:01 and DQB1*04:02, as these alleles strongly bound this α-synuclein epitopes^19^. These HLA alleles, however, are not associated with the risk of PD in the current study. However, the authors reported in the supplementary data that DRB1*04:01 was also a selective and strong binder of the same α-synuclein epitope with p.S129, but only when the epitope was unphosphorylated^19^. Notably, no other DRB1 alleles that were assayed in this study^19^ had increased binding affinity to the α-synuclein epitope with p.S129, except for the DRB1*04:01 allele that was a strong binder only when unphosphorylated. Binding register analysis using Immune Epitope Database (IEDB) MHC-II Binding Prediction (http://tools.iedb.org/mhcii/) suggests that this epitope binds a 9 amino acid AYEMPSEEG core, with p.S129 at the P6 position, a position postulated to be important based on our HLA-DR amino acid analysis presented above. As CD4+ T cell responses are generally stronger when epitopes are presented by HLA-DR vs. HLA-DQ^23^, HLA-DRB1*04 responses to the p.S129 unphosphorylated form of α-synuclein could be dominant in individuals with HLA-DRB1*04, explaining the protective effect of this HLA subtype in PD. Additional experiments will be needed to further explore this hypothesis.

Our study has several limitations. First, this study was performed on European populations, and the results may be limited to this population only. Additional studies in other populations are required. Several studies on *HLA* types and PD have been performed in Asian populations^24–27^, and the GWAS risk variant rs112485576 has a similar OR (0.85) in the largest Asian GWAS to date^28^, yet larger studies are required, as well as studies in other populations. An additional potential limitation of our study is its use of imputation rather than fully sequenced HLA types. Given the very high performance of the imputation tool when compared to full sequencing (Supplementary Data 7), the potential effect of imputation inaccuracies is likely small and should be diluted in our large sample size. Since different cohorts may have had different criteria for determining the presence of PD, it is important to note that for all the positive associations reported in the current study, the directionality was identical across all cohorts with similar effect magnitude, and for all meta-analyses the heterogeneity was not statistically significant (supplementary Data 3–5), indicating that the different criteria in the different cohorts did not have a major effect on the results. In addition, we cannot rule out that other, rarer HLA types that were not included in the current analysis may also have a role in PD. An additional limitation of the current study is that by adjusting for sex we eliminate potential sex-specific effects. It is possible that specific HLA types are relevant in one sex more or less than the other, and this should be studied in larger, sex-stratified cohorts.

To conclude, our results suggest a role for the *HLA-DRB1* gene in susceptibility for PD and provide further evidence for the importance of the immune system in PD. Since the effect is small, it does not merit routine HLA typing in PD, but understanding the mechanism underlying this association may lead to a better understanding of PD in general and offer new targets for future immune-related treatment.

## Methods

### Study population

This study was designed as a meta-analysis of multiple cohorts, including a total of 13,770 PD patients, 20,214 proxy-patients, and 490,861 controls, as detailed in Supplementary Data 1 and 2. In brief, we included cohorts and datasets from eight independent sources: International Parkinson’s Disease Genomics Consortium (IPDGC) NeuroX dataset (dbGap phs000918.v1.p1, including datasets from multiple independent cohorts as previously described)^29^, McGill University (McGill)^30^, National Institute of Neurological Disorders and Stroke (NINDS) Genome-Wide genotyping in Parkinson’s Disease (dbGap phs000089.v4.p2)^31^, NeuroGenetics Research Consortium (NGRC) (dbGap phs000196.v3.p1)^11^, Oslo Parkinson’s Disease Study (Oslo), Parkinson’s Progression Markers Initiative (PPMI), Vance (dbGap phs000394) and PD cases and proxy-cases from the UK Biobank (UKB). Proxy-cases are first-degree relatives of PD patients, thus sharing ~50% of the patients’ genetic background and eligible to serve as proxies, as previously described^32^. Since they are genetically very similar, although they do not have PD, each proxy case represents a case of PD, allowing us to increase the power. However, we also analyzed the data without the proxy-cases cohort to make sure that they did not introduce any bias. All cohorts were previously included in the most recent PD GWAS^6^. Study protocols were approved by the Institutional Review Board at McGill University and all patients signed informed consent before participating in the studies.

### Pre-imputation genotype quality control

In order to include only high-quality samples and SNPs, standard quality control (QC) was performed on all datasets individually using PLINK v1.9^33^. Standard GWAS QC was done to filter out samples and SNPs with low call rates, heterozygote outliers along gender mismatch as previously described^6^. SNPs deviating from Hardy-Weinberg equilibrium were removed. Only samples of European ancestry clustering with HapMap v3 using principal component analysis were included as shown in Supplementary Fig. 5. In order to exclude related individuals, we examined relatedness in each dataset separately, followed by a relatedness test across all datasets combined, to exclude individuals who were included in more than one dataset. All individuals with pi_hat >0.125 were excluded using GCTA v1.26.0^34^.

### UK Biobank QC

For the analysis of the UKB data, unrelated participants of European ancestry (field 22006), with a low missingness rate (field 220027) were included after the exclusion of heterozygosity outliers as previously described^6^. PD patients from the UK Biobank were included based on self-report (field 20002) or based on their International Classification of disease diagnosis code (ICD-10, code G20, field 41270). From the remaining participants, proxy-cases were defined as first-degree relatives (parents or siblings, field 20112–20114) of patients with PD. Principal components were calculated using flashpca^35^ after excluding related individuals as described above. The control group was divided randomly into two groups of controls: one was included in the GWAS comparing PD patients from UKB and controls, and the second was included in the GWAS comparing proxy-cases from UKB and controls. This division was done proportionally to the size of each GWAS.

### Imputation

For the SNP imputation of each dataset, we used the Michigan Imputation Server on the 1000 Genomes Project panel (Phase 3, Version 5) using Minimac3 and SHAPEIT v2.r790. Imputed UK Biobank genotyped data v3 were downloaded in July 2019. All variants with an imputation quality (*r*^*2*^) of >0.30 were labeled as soft calls and >0.80 were labeled as hard calls. Soft calls were only used together with the hard calls for polygenic risk score (PRS) calculation (see below); hard calls were used for all other analyses.

### Association analysis of common variants on chromosome 6

Prior to determining HLA types, we performed a simple association test of all SNPs located on chromosome 6, to verify that we identified the same hit in the HLA region as previously described^6^. For this purpose, we generated summary statistics of chromosome 6 for each dataset and used logistic regression with an additive model adjusting for age at onset for patients and age at enrollment of controls, sex, and population stratification (first 10 principal components) with PLINK v2.00a2LM (25 Oct 2019)^33^. The UK Biobank data were analyzed similarly using logistic regression adjusting for age, sex, the first 10 principal components, and Townsend index to account for additional potential population stratification confounders. Finally, to harmonize effects in cases and proxy cases, summary statistics for proxy cases were rescaled based on genome-wide association study by proxy as previously described^32^. Each dataset was analyzed separately, followed by a meta-analysis of all datasets. To meta-analyze the different datasets, we performed a fixed-effects meta-analysis using METAL with an inverse-variance-based model and examined whether heterogeneity exists between the different cohorts^36^.

### HLA locus analysis

To impute specific HLA types for each individual, we inferred two field resolution HLA alleles using HIBAG v1.22.0, a statistical method for HLA type imputation in R^37^. HIBAG was shown to be as accurate or more accurate in Europeans compared to other types of HLA imputation tools^38^. HIBAG provided a reference panel for Europeans (*n* = 2572) with high imputation accuracy for *HLA-A*, *HLA-B*, *HLA-C*, class I genes, and *HLA-DPB1*, *HLA-DQA1*, *HLA-DQB1*, and *HLA-DRB1*, class II genes. *HLA-DRB3*, *HLA-DRB4*, and *HLA-DRB5* imputation models were trained using HIBAG^37^ on European origin sample training set (*n* = 3267) genotyped on the Illumina Infinium PsychArray-24 chip and fully sequenced at 8-digit resolution for HLA loci. These models were validated in a test set (*n* = 886) with high accuracy (Supplementary Data 7). Imputation accuracy for European DRB1*04 alleles was determined for DRB1*04:01 DRB1*04:02 DRB1*04:03, DRB1*04:04, DRB1*04:05, DRB1*04:07, DRB1*04:08. Alleles with an imputation probability of <0.5 were defined as undetermined and individuals with two or more undetermined alleles were excluded from the analysis (Supplementary Data 1 details the numbers included for each allele in each cohort after all quality control steps). To further examine imputation accuracy, the results of the *DRB1* imputation were compared against high throughput HLA sequencing in 380 PD samples from Oslo. The combined frequency of seven different DRB1*04 alleles detected in sequence data was 0.15 with the 04:01 and 04:04 alleles being the most common (Supplementary Data 8). Imputation accuracy for DRB1*04 alleles was very high at 2-digit resolution (Supplementary Data 9).

To examine the association of HLA alleles with PD, we used R v3.6 to perform logistic regression, adjusting for age at onset, sex, and the first 10 principal components. The UK Biobank dataset was also adjusted for the Townsend index. Haplotype analyses were performed using haplo.stats in R with logistic regression as stated above. Only haplotypes with posterior probability >0.2 and a carrier frequency of >1% were included in the analysis. Amino acid association analyses were performed using HIBAG after converting P-coded alleles to amino acid sequences for exon 2, 3 of HLA class I genes, and exon 2 of class II genes. Amino acid associations were tested using logistic regression as described above. A PRS was calculated using PRSice v 2.2.11 without linkage disequilibrium (LD) clumping or P thresholding^39^. The beta weights from the summary statistics of the 90 genome-wide significant variants in the latest PD GWAS^6^ were used in the PRS. To make sure that all possible variants were included in the PRS analysis, we also performed imputation using the Haplotype Reference Consortium panel (Version r1.1 2016) with Minimac4 and Eagle v2.4. Ambiguous variants (rs6658353) and rs112485576 from the HLA region were excluded from the PRS calculation. We also used the top hit in the HLA region from this GWAS to perform adjustment in the regression models and conditional analyses to determine whether the associations reported here are in LD with the GWAS top hit. To examine whether secondary hits exist in the HLA region, we adjusted for significant HLA variants, HLA alleles, HLA amino acid changes, and PRS, by introducing significant findings from the first analyses as covariates in the regression models. Statistical analyses were only performed on alleles, haplotypes, and amino acid changes with more than 1% carrier frequency. *P* value significance levels were adjusted using Bonferroni correction. Meta-analysis was performed as described above. All missing data were excluded from the analyses.

### Reporting summary

Further information on research design is available in the Nature Research Reporting Summary linked to this article.

## Supplementary information

Supplementary Information

Supplementary Data 1

Supplementary Data 2

Supplementary Data 3

Supplementary Data 4

Supplementary Data 5

Supplementary Data 6

Supplementary Data 7

Supplementary Data 8

Supplementary Data 9

Reporting Summary

## Data Availability

Anonymized data will be shared by request from any qualified investigator. All summary statistics are found in the Supplementary Data. The accession numbers to the datasets from dbGaP (https://www.ncbi.nlm.nih.gov/gap/) used in this study are: International Parkinson’s Disease Genomics Consortium (IPDGC) NeuroX dataset (dbGap phs000918.v1.p1), National Institute of Neurological Disorders and Stroke (NINDS) Genome-Wide genotyping in Parkinson’s Disease (dbGap phs000089.v4.p2), NeuroGenetics Research Consortium (NGRC) (dbGap phs000196.v3.p1) and Vance (dbGap phs000394).

## Code availability

The scripts used in this analysis is available at https://github.com/gan-orlab/HLA_HIBAG/.
