# Supplementary material for: Fine mapping of the HLA locus in Parkinson’s disease in Europeans
Source: NPJ Parkinsons Dis. 2021 Sep 21;7:84. doi: 10.1038/s41531-021-00231-5 (PMC8455634; doi:10.1038/s41531-021-00231-5)
Supplement: Supplementary file 1 — Supplementary Information [file 41531_2021_231_MOESM1_ESM.pdf]

Supplementary Information

Fine mapping of the *HLA* locus in Parkinson's disease in Europeans

Eric Yu, BSc,[1,2] Aditya Ambati, PhD,[3] Maren Stolp Andersen, MD,[4,5] Lynne Krohn, MSc,[1,2] Mehrdad A. Estiar, MSc,[1,2] Prabhjyot Saini, MSc,[1,2] Konstantin Senkevich, MD, PhD,[2,6] Yuri L. Sosero, MD,[1,2] Ashwin Ashok Kumar Sreelatha, MSc, MTech,[7] Jennifer A. Ruskey, MSc,[2,6] Farnaz Asayesh, MSc,[2,6] Dan Spiegelman, MSc,[2,6] Mathias Toft, MD, PhD,[4,5] Marte K. Viken, PhD,[8,9] Manu Sharma, PhD,[7] Cornelis Blauwendraat, PhD,[10] Lasse Pihlstrøm, MD, PhD,[4] Emmanuel Mignot, MD, PhD*,[3] Ziv Gan-Or, MD, PhD*.[1,2,6]

Supplementary Figure 1

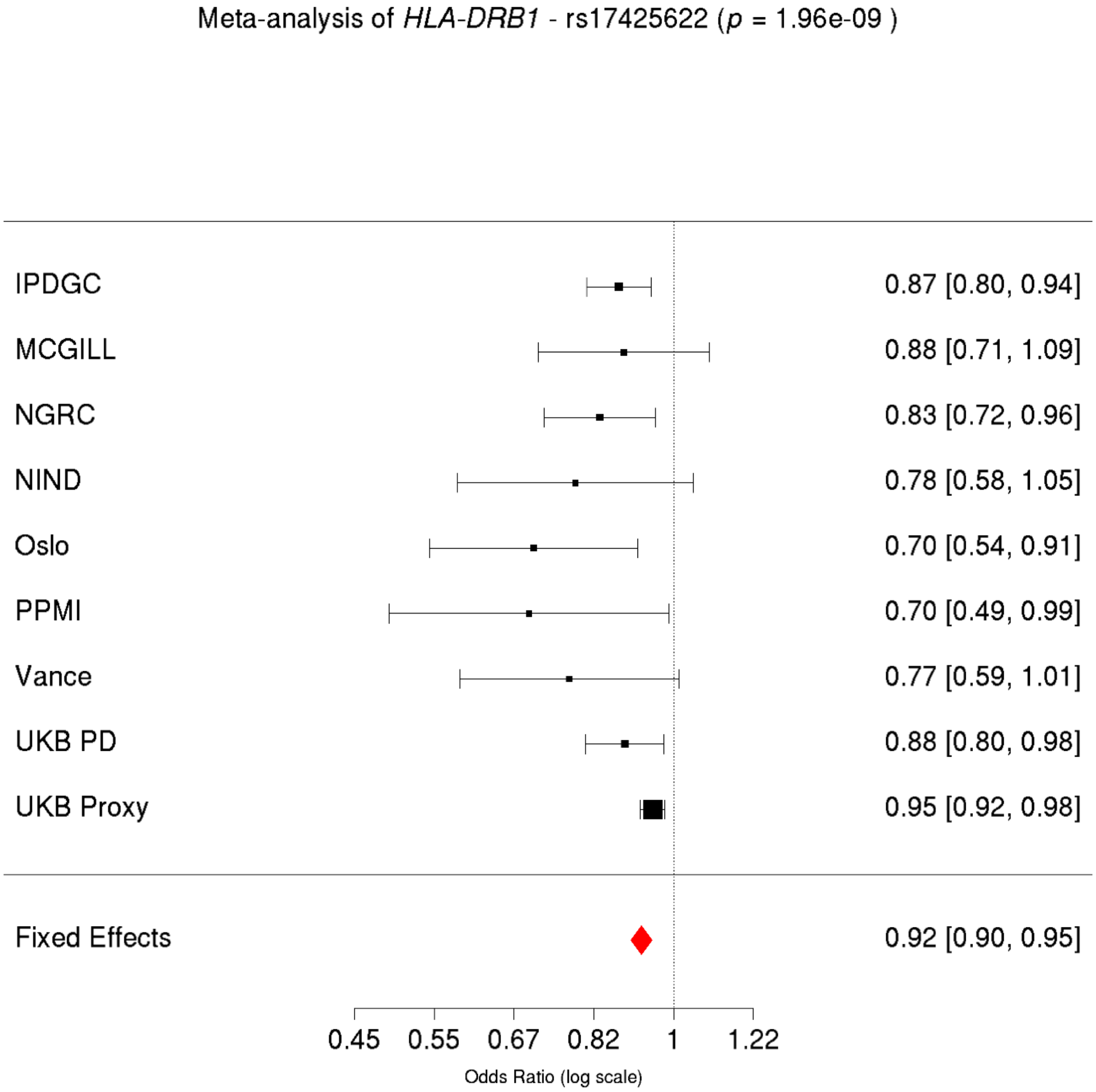

Supplementary Figure 1:

A forest plot depicting the meta-analysis of the *HLA-DRB1* rs17425622 SNP in Parkinson's disease, showing a similar directionality across all cohorts and association with reduced risk of Parkinson's disease in the meta-analysis (OR = 0.92, 95% CI 0.90-0.95, *p*=1.96e-9).

Supplementary Figure 2

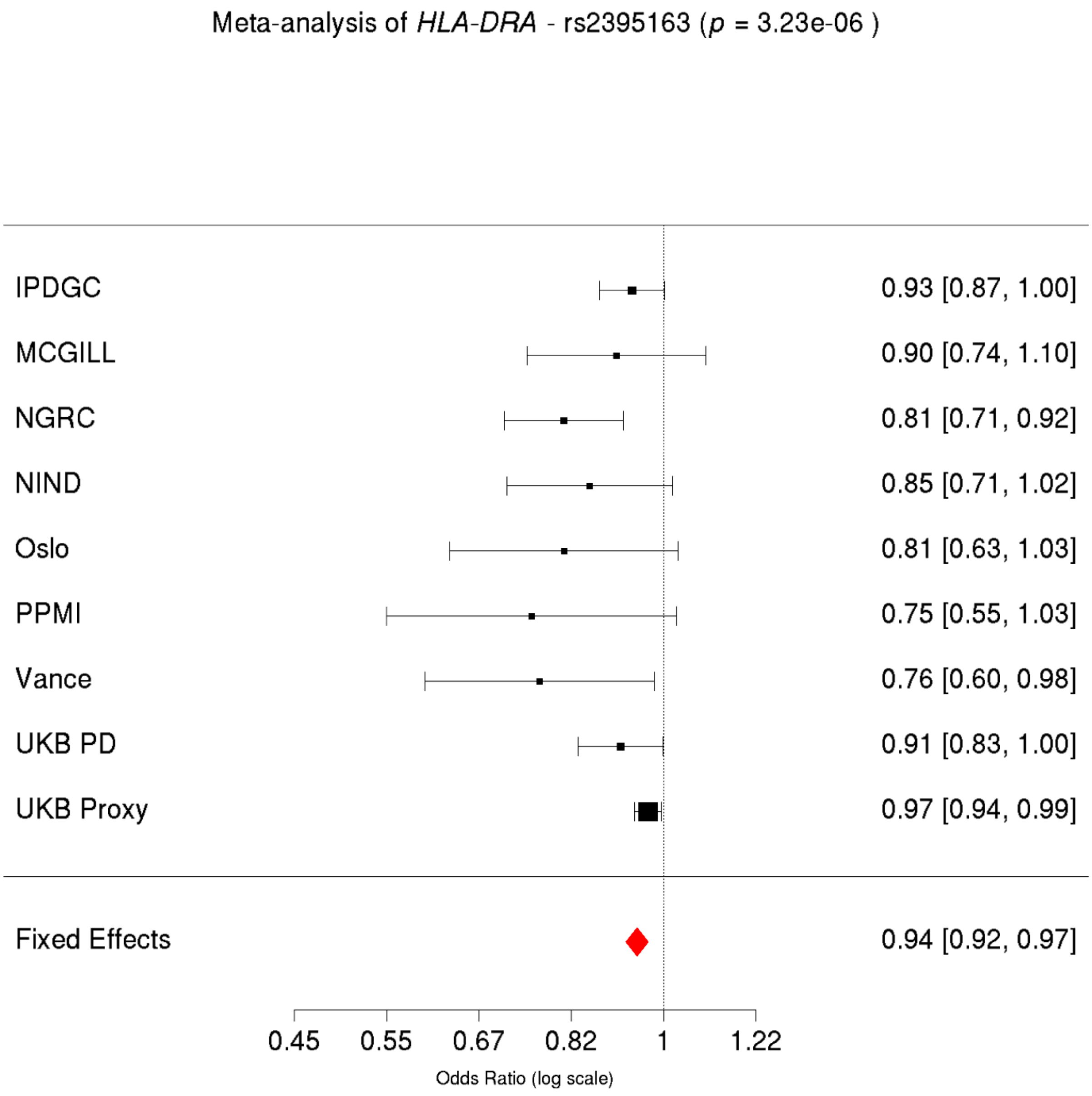

Supplementary Figure 2:

A forest plot depicting the meta-analysis of the *HLA-DRA* rs2395163 SNP in Parkinson's disease, showing a similar directionality across all cohorts and association with reduced risk of Parkinson's disease in the meta-analysis (OR = 0.94, 95% CI 0.92-0.97, *p*=3.23e-6).

Supplementary Figure 3

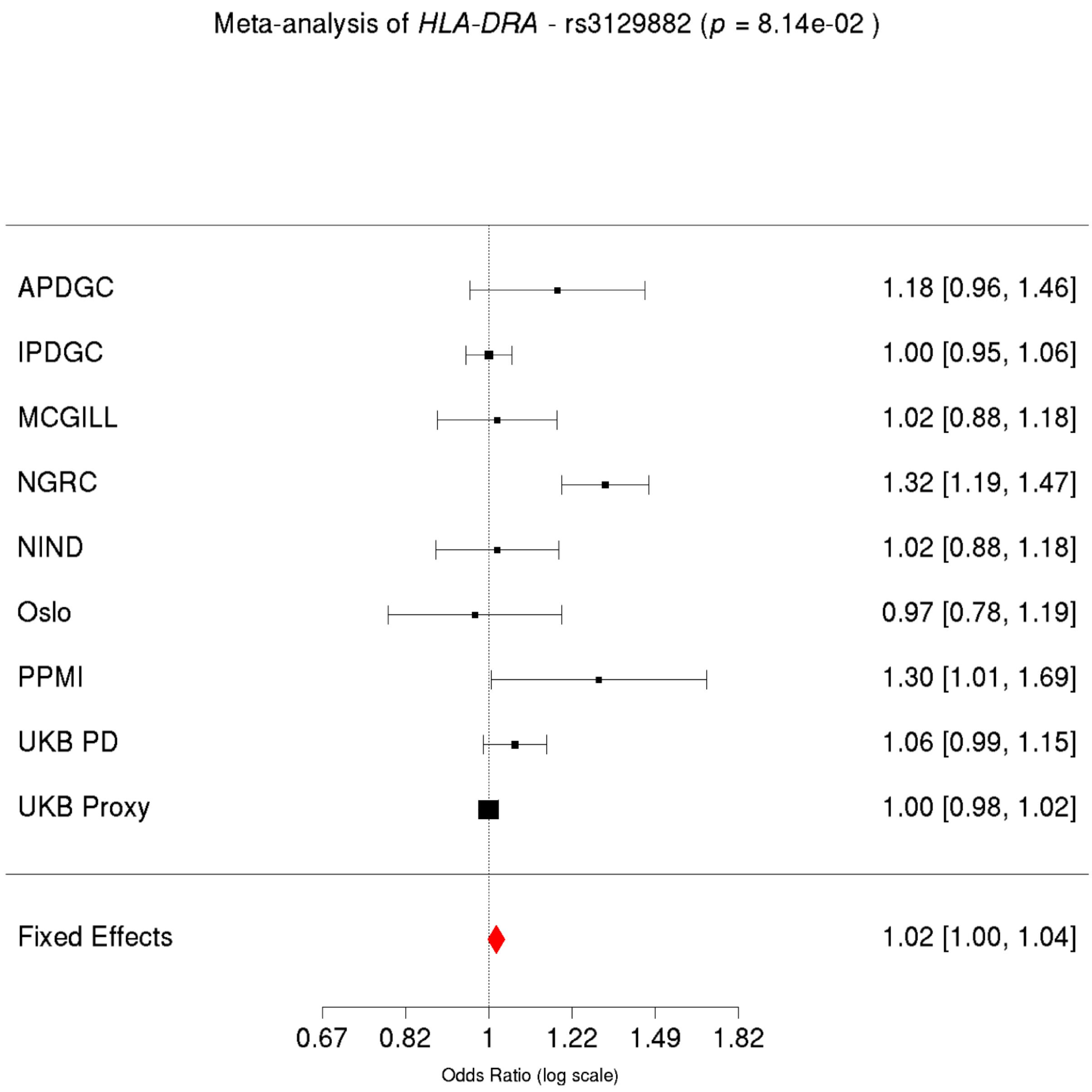

Supplementary Figure 3:

A forest plot depicting the meta-analysis of the *HLA-DRA* rs3129882 SNP in Parkinson's disease, showing lack of association with Parkinson's disease in the meta-analysis (OR = 1.02, 95% CI 1.00-1.04, *p*=0.0814).

Supplementary Figure 4

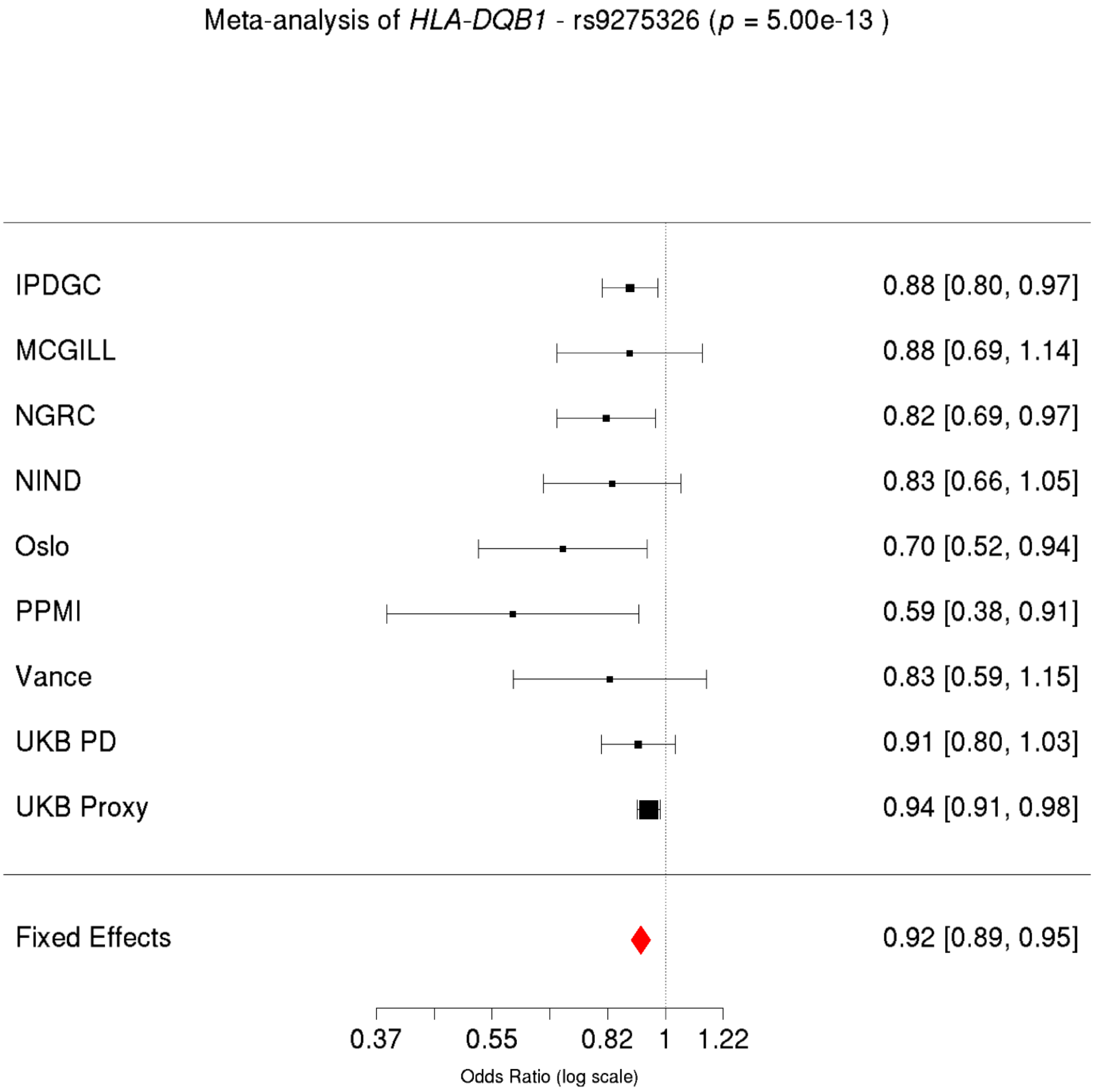

Supplementary Figure 4:

A forest plot depicting the meta-analysis of the *HLA-DQB1* rs9275326 SNP in Parkinson's disease, showing a similar directionality across all cohorts and association with reduced risk of Parkinson's disease in the meta-analysis (OR = 0.92, 95% CI 0.89-0.95, *p*=5.00e-13).

Supplementary Figure 5

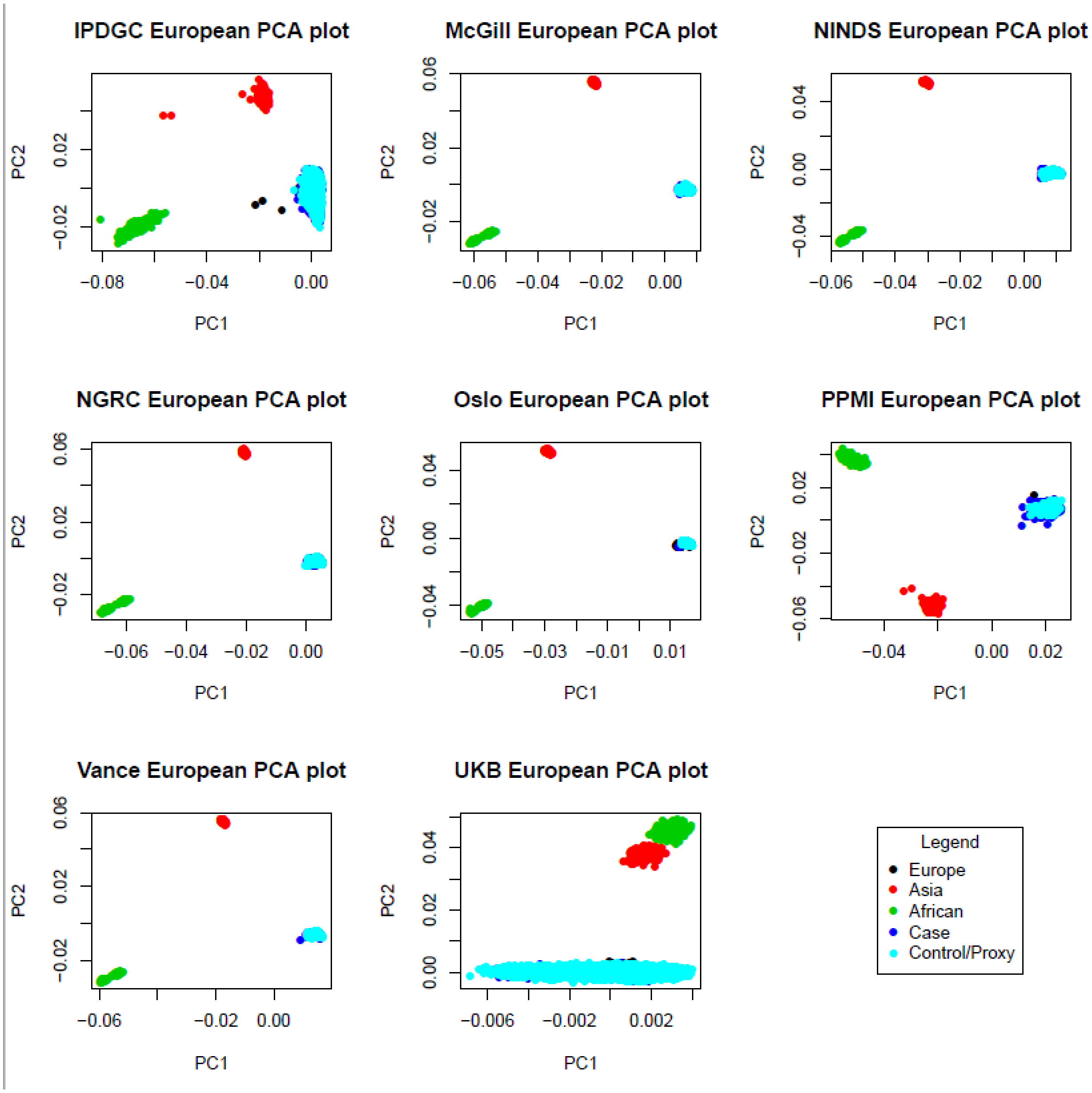

Supplementary Figure 5:

Principal component analysis demonstrating the overlap of cases and controls used in the different cohorts in the current study with individuals of European ancestry.

Supplementary Data:

This manuscript contains nine Supplementary Datasets, including:

Supplementary Dataset 1: Table describing the populations used for the different analysis phases - Total number of cases and controls are shown for each step of the analysis. In "Sequenced", the total number of samples sequenced. In "Standard QC", the total numbers of samples which passed the quality control phase. In "HLA allele QC", the total numbers of samples which passed the quality control phase for HLA alleles. In "HLA allele adjusted", the total numbers of samples for any conditional analysis. In "HLA haplotype", the total numbers of samples after quality control for haplotype analysis. In "HLA amino acid", the total numbers of samples after quality control for amino acid association.

Supplementary Dataset 2: Table providing basic demographic details on the participating cohorts.

Supplementary Dataset 3: Results of the meta-analyses of different HLA allele association with PD sorted by gene, then by p-value.

Supplementary Dataset 4: Results of the meta-analyses of different class II HLA haplotype association with PD sorted by gene, then by p-value.

Supplementary Dataset 5: Results of the meta-analyses of different HLA amino acid association with PD sorted by gene, then by p-value.

Supplementary Dataset 6: Results on previously reported HLA hits in Parkinson's disease in the current study.

Supplementary Dataset 7: Table describing frequencies and accuracy of imputation in a test set of 3,267 individuals with full sequencing data of DRB3, DRB4 and DRB5. "valid.num" - the number of each detected allele; "valid.freq" - the frequency of each detected allele; "ppv" - positive predictive value; "npv" - negative predictive value".

Supplementary Dataset 8: Allele frequency of imputed HLA-DRB1*04 using HIBAG in Oslo patients.

Supplementary Dataset 9: Sensitivity and Specificity of imputed HLA-DRB1*04 using HIBAG in Oslo patients.
