# Supplementary material for: Fine mapping of the HLA locus in Parkinson’s disease in Europeans
Source: NPJ Parkinsons Dis. 2021 Sep 21;7:84. doi: 10.1038/s41531-021-00231-5 (PMC8455634; doi:10.1038/s41531-021-00231-5)
Supplement: Supplementary file 11 — Reporting Summary [file 41531_2021_231_MOESM11_ESM.pdf]

Corresponding author(s): Ziv Gan-Or

Last updated by author(s): Jun 28, 2021

Reporting Summary

Nature Portfolio wishes to improve the reproducibility of the work that we publish. This form provides structure for consistency and transparency in reporting. For further information on Nature Portfolio policies, see our Editorial Policies and the Editorial Policy Checklist.

Statistics

For all statistical analyses, confirm that the following items are present in the figure legend, table legend, main text, or Methods section.

n/a	Confirmed	
☐	☒	The exact sample size (n) for each experimental group/condition, given as a discrete number and unit of measurement
☐	☒	A statement on whether measurements were taken from distinct samples or whether the same sample was measured repeatedly
☐	☒	The statistical test(s) used AND whether they are one- or two-sided<br>*Only common tests should be described solely by name; describe more complex techniques in the Methods section.*
☐	☒	A description of all covariates tested
☐	☒	A description of any assumptions or corrections, such as tests of normality and adjustment for multiple comparisons
☐	☒	A full description of the statistical parameters including central tendency (e.g. means) or other basic estimates (e.g. regression coefficient) AND variation (e.g. standard deviation) or associated estimates of uncertainty (e.g. confidence intervals)
☐	☒	For null hypothesis testing, the test statistic (e.g. F, t, r) with confidence intervals, effect sizes, degrees of freedom and P value noted<br>*Give P values as exact values whenever suitable.*
☒	☐	For Bayesian analysis, information on the choice of priors and Markov chain Monte Carlo settings
☒	☐	For hierarchical and complex designs, identification of the appropriate level for tests and full reporting of outcomes
☒	☐	Estimates of effect sizes (e.g. Cohen's d, Pearson's r), indicating how they were calculated

Our web collection on statistics for biologists contains articles on many of the points above.

Software and code

Policy information about availability of computer code

Data collection	No software was used for data collection
Data analysis	All code used for data analysis can be found at https://github.com/gan-orlab/HLA_HIBAG/

For manuscripts utilizing custom algorithms or software that are central to the research but not yet described in published literature, software must be made available to editors and reviewers. We strongly encourage code deposition in a community repository (e.g. GitHub). See the Nature Portfolio guidelines for submitting code & software for further information.

Data

Policy information about availability of data

All manuscripts must include a data availability statement. This statement should provide the following information, where applicable:

- Accession codes, unique identifiers, or web links for publicly available datasets
- A description of any restrictions on data availability
- For clinical datasets or third party data, please ensure that the statement adheres to our policy

Anonymized data will be shared by request from any qualified investigator

Field-specific reporting

Please select the one below that is the best fit for your research. If you are not sure, read the appropriate sections before making your selection.

☒ Life sciences ☐ Behavioural & social sciences ☐ Ecological, evolutionary & environmental sciences

For a reference copy of the document with all sections, see nature.com/documents/nr-reporting-summary-flat.pdf

Life sciences study design

All studies must disclose on these points even when the disclosure is negative.

Sample size	Sample size was achieved by collecting all the publicly available genetics datasets of Parkinson's disease patients and controls of European Ancestry
Data exclusions	Data was excluded based on different QC thresholds:<br>In order to include only high-quality samples and SNPs, standard quality control (QC) was performed on all datasets individually using PLINK v1.9.33 Standard GWAS QC was done to filter out samples and SNPs with low call rate, heterozygote outliers along with gender mismatch as previously described.6 SNPs deviating from Hardy-Weinberg equilibrium were removed. Only samples of European ancestry clustering with HapMap v3 using principal component analysis were included as shown in Supplementary Figure 5. In order to exclude related individuals, we examined relatedness in each dataset separately, followed by relatedness test across all datasets combined, to exclude individuals who were included in more than one dataset. All individuals with pi_hat >0.125 were excluded using GCTA v1.26.0.<br>For the analysis of the UKB data, unrelated participants of European ancestry (field 22006), with low missingness rate (field 220027) were included after exclusion of heterozygosity outliers as previously described.6. PD patients from the UK Biobank were included based on self-report (field 20002) or based on their International Classification of disease diagnosis code (ICD-10, code G20, field 41270). From the remaining participants, proxy-cases were defined as first degree relatives (parents or siblings, field 20112-20114) of patients with PD. Principal components were calculated using flashpca35 after excluding related individuals as described above. The control group was divided randomly to two groups of controls: one was included in the GWAS comparing PD patients from UKB and controls, and the second was included in the GWAS comparing proxy-cases from UKB and controls.<br>For SNP imputation of each dataset, we used the Michigan Imputation Server on the 1,000 Genomes Project panel (Phase 3, Version 5) using Minimac3 and SHAPEIT v2.r790. Imputed UK Biobank genotyped data v3 were downloaded in July 2019. All variants with an imputation quality (r2) of >0.30 were labeled as soft calls and >0.80 were labeled as hard calls. Soft calls were only used together with the hard calls for polygenic risk score calculation (see below); hard calls were used for all other analyses.
Replication	This is a meta-analysis, no additional replication was performed.
Randomization	Samples were defined as patients if they were diagnosed with Parkinson's disease and as controls if they were not. Adjustment for age, sex and principal components of ancestry was performed as needed.
Blinding	Not relevant to the study, which is a genetic case-control study

Reporting for specific materials, systems and methods

We require information from authors about some types of materials, experimental systems and methods used in many studies. Here, indicate whether each material, system or method listed is relevant to your study. If you are not sure if a list item applies to your research, read the appropriate section before selecting a response.

Materials & experimental systems

n/a	Involved in the study	
☒	☐	Antibodies
☒	☐	Eukaryotic cell lines
☒	☐	Palaeontology and archaeology
☒	☐	Animals and other organisms
☐	☒	Human research participants
☒	☐	Clinical data
☒	☐	Dual use research of concern

Methods

n/a	Involved in the study	
☒	☐	ChIP-seq
☒	☐	Flow cytometry
☒	☐	MRI-based neuroimaging

Human research participants

Policy information about studies involving human research participants

Population characteristics	This is described in details in the paper and in the supplementary tables.
Recruitment	Participants were recruited to different cohorts in previous studies, and we have collected publicly available data on them.

Ethics oversight	Each of the studies from which data were collected was approved by their respective IRB.

Note that full information on the approval of the study protocol must also be provided in the manuscript.
